# Effect of Antigravity Treadmill Training on Gait and Balance in Patients with Diabetic Polyneuropathy: A Randomized Controlled Trial

**DOI:** 10.12688/f1000research.75806.3

**Published:** 2022-10-26

**Authors:** Ashraf Abdelaal, Shamekh El-Shamy

**Affiliations:** 1 Physical Therapy, Umm-Al-Qura University, Makkah, 715, Saudi Arabia

**Keywords:** Diabetic polyneuropathy, antigravity treadmill, traditional rehabilitation, gait, balance

## Abstract

**Background:** Diabetic polyneuropathy (DPN) is the most prevalent consequence of diabetes mellitus, and it has a significant impact on the patient's health. This study aims to evaluate effects of antigravity treadmill training on gait and balance in patients with DPN. **Methods: **The study included 45 males with type 2 diabetes who were randomly assigned to one of two groups: the experimental group (n=23) or the control group (n=22). For a period of 12 weeks, the experimental group received antigravity treadmill training (75% weight bearing, 30 min per session, three times per week) combined with traditional physical therapy. During the same time period, the control group received only traditional physical therapy. The Biodex Balance System was used to assess postural stability indices, while the GAITRite Walkway System was used to assess spatiotemporal gait parameters. All measurements were obtained before and at the end of the study after 12 weeks of treatment. **Results: **The mean values of all measured variables improved significantly in both groups (P<0.05), with the experimental group showing significantly greater improvements than the control group. The post-treatment gait parameters ( *i.e.*, step length, step time, double support time, velocity, and cadence) were 61.3 cm, 0.49 sec, 0.25 sec, 83.09 cm/sec, and 99.78 steps/min as well as 56.14 cm, 0.55 sec, 0.29 sec, 75.73 cm/sec, and 88.14 steps/min for the experimental and control group, respectively. The post-treatment overall stability index was 0.32 and 0.70 for the experimental and control group, respectively. **Conclusions:** Antigravity treadmill training in combination with traditional physical therapy appears to be superior to traditional physical therapy alone in terms of gait and balance training. As a result, the antigravity treadmill has been found to be an effective device for the rehabilitation of DPN patients.

## Introduction

Diabetes mellitus (DM) is a long-term metabolic disorder that impairs the body's capacity to control blood glucose levels. DM is a major health concern, with an increase in the prevalence of type 2 DM (T2DM), which has been linked to lifestyle factors. Long-term microvascular and macrovascular complications are responsible for the disease's high morbidity and mortality, and they place patients and healthcare systems under a great deal of stress. ^1^

Diabetic polyneuropathy (DPN) is the most common complication. It's linked to having diabetes for a long time and having poor glycemic control, which causes a lot of pain and distress. Saudi Arabia is the largest country in the Middle East, occupying approximately four-fifths of the Arabian Peninsula and residences to over 33.3 million people. Diabetes affects approximately one-fourth of people above the age of 30. ^2^

DPN is a progressive degeneration of the peripheral nerves that affects the sensory, motor, and autonomic components of the nerves, causing loss of protective sensation, intrinsic foot muscle dysfunction, and foot anhydrosis. ^3^ It leads to increased public health costs and has a significant impact on patient quality of life when not treated properly. Plantar ulceration and amputation, the disease's most fatal outcome, can still be avoided if proper measures are applied. ^4^

DPN affects the quantity and quality of sensory information used in gait generation and control, causing alterations in the sensory and motor control systems. Reduced range of motion, muscle strength, and changes in gait parameters are all linked to reduced mobility and impaired balance. ^5^ In comparison to healthy controls, DPN patients exhibit a lower gait velocity, lower cadence, shorter stride length, longer stance time, and higher steps variability. These alterations in gait are more noticeable when walking on uneven surfaces. DPN patients exhibit a lower ankle moment and ankle power, as well as a different onset and cessation time of muscle activity than healthy controls. ^6^

Despite the fact that current physical therapy therapies can help patients with DPN improve their gait and balance, the effects are minor. ^7^ In two systematic studies of DPN therapies, lower extremity strengthening was only given a fair recommendation. ^8^^,^^9^ Other pain-relieving and function-improving therapies, such as electrotherapy and monochromatic light therapy, lacked sufficient evidence to be recommended. ^9^ Salsabili *et al.* ^10^ showed that following four weeks of task-oriented gait and balance training, Timed "Up and Go" scores and Falls Efficacy Scale scores improved.

The antigravity treadmill is a unique method of preserving a patient's body weight during treadmill training that was recently developed. An inflatable treadmill is included in this system. The patient is dressed in neoprene shorts that are zipped up within the bag. The air pressure in the bag, which acts as a lifting force on the body, determines how much body weight needs to be supported. The air pressure is equally distributed across the lower body, minimizing the pressure points found in traditional body weight support systems. ^11^

Abdelaal and El-shamy, ^12^ concluded in a recent trial that a moderate intensity antigravity treadmill training can significantly improve gait, balance, and fall risk scores in patients with DPN. During this moderate intensity aerobic training in patients with DPN, the 75% weight bearing had more significant results than the 0 %, 25%, and 50% weight bearing on gait, balance, and fall risk scores. Therefore, using the lower body positive pressure (LBPP) technology for unweighting patients with DPN during antigravity treadmill training can provide a new treatment modality for patients with DPN.

Despite the growing popularity of LBPP treadmills, it's unclear how valid the available scientific evidence to support their use in the rehabilitation of patients with DPN. The purpose of this study was therefore to investigate the effects of antigravity treadmill training on gait and balance in patients with DPN.

## Methods

### Design

This single-blind randomized controlled trial was designed to study the effects of antigravity treadmill training on gait and balance in patients with DPN. It was approved by the Ethical Committee of the Faculty of Applied Medical Sciences, Umm Al-Qura University (15-MED5221-10). This trial was registered in the ClinicalTrial.gov PRS No NCT05088993. Before signing an informed consent form accepting to participate in the study, all participants were given a full explanation of the study's procedures, hazards, and objectives.

### Participants

45 elderly Saudi men diagnosed with DPN are recruited from Umm Al-Qura University Medical Center, Makkah, Saudi Arabia, and referred to a physiotherapist, who performs the initial assessment. The inclusion criteria for this study were age between 60 to 80 years old; having uncontrolled T2DM more than 10 years, with DPN; glycosylated hemoglobin (HbA1c %) level between 7–11%, fasting blood glucose level of 7.0–11.1 mmol/L; treated only with oral hypoglycemic medications; able to walk independently with or without assistive devices; with normal nutritional status; cognitively competent and able to understand and follow instructions.

The exclusion criteria were patients with type 1 diabetes; younger than 60 or older than 80 years old; following diet regimen; patients with malnutrition (BMI<21 kg/m ^2^ or with recent weight loss >5% body weight in the last month or >10% in six months); recently involved in an exercise training program within the last six months; underwent surgical intervention within the last six months; patients with feet ulcers; patients with serious cardiovascular insult or sever complications that can impact patient's safety, performance and affects study outcomes.

### Sample-size calculation

The proper sample size was calculated using G*Power for Windows (G*Power 3.1.3, RRID:SCR_013726) with estimated power (1-error probability) = 0.95, = 0.05. The effect size was 0.8 using unpaired t-test. A minimum of 40 patients was specified as a sample size for this study. An extra five participants were added above the required number to compensate any potential dropouts.

### Randomization

**Figure 1.**
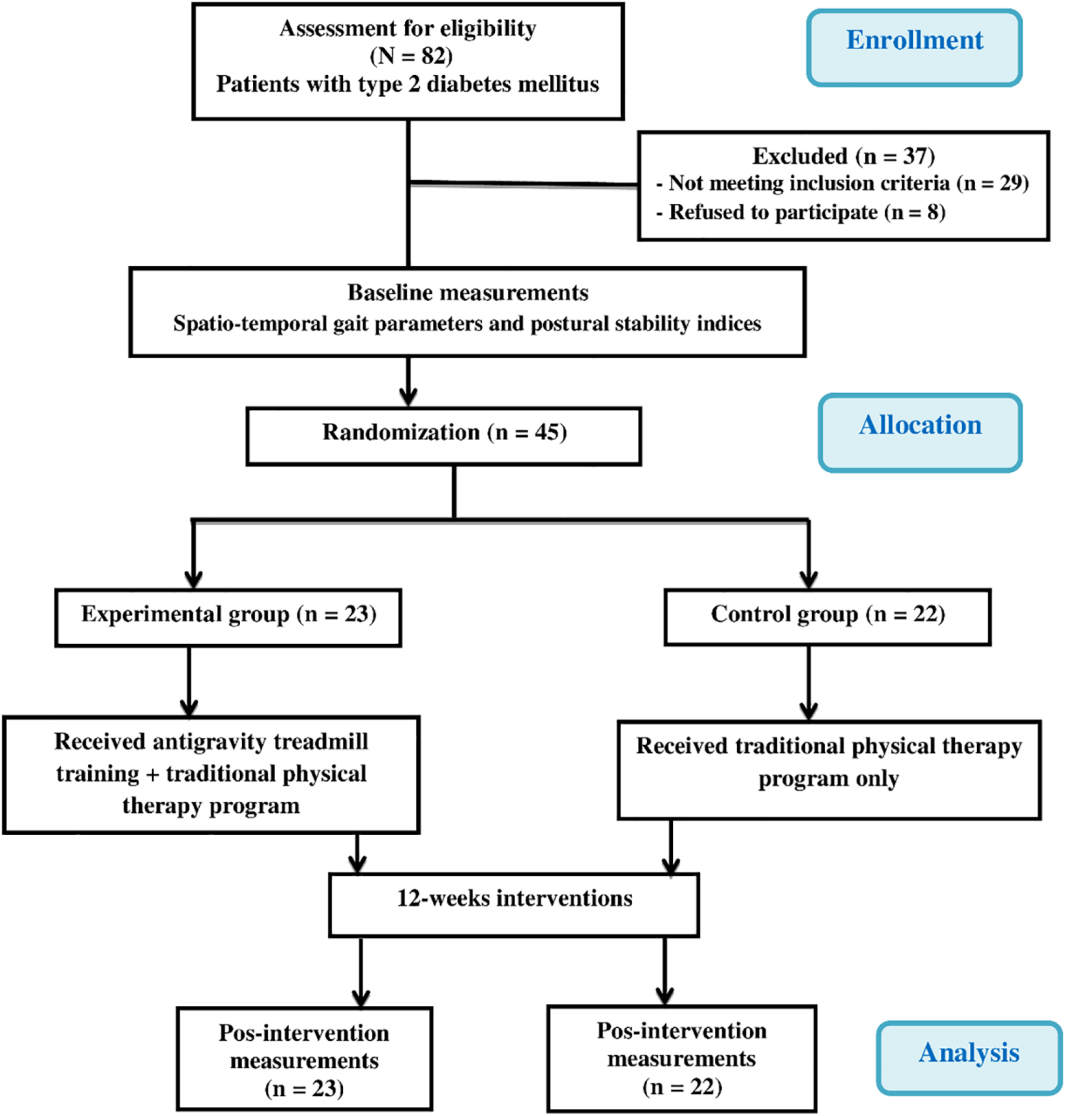
Flow chart showing the experimental design of the study.

Patients were randomly assigned to experimental (n = 23) and control (n = 22) therapy groups using an online randomization website ( www.randomization.com) to prevent bias in the treatment assignment. Traditional physical therapy was delivered to the control group. The experimental group, on the other hand, received antigravity treadmill training in addition to the traditional program given to the control group. Both groups received the usual medical care during the study period. The therapists who performed the measurements and evaluated the results were blind of the groups' assignments. Figure 1 demonstrates the experimental design as a flow chart.

### Outcome measures

Patients’ demographic data and clinical characteristics including age and diabetes duration in years were recorded, weight in kg and height in meter, body mass index (BMI; kg/m ^2^), blood pressure (BP) in mmHg, and resting heart rate (HR). fasting blood glucose (FBG) level and glycosylated hemoglobin % (HbA1c) were evaluated according to the American Diabetes Association guidelines. Evaluation of the DPN was done using the Michigan neuropathy screening instrument. Nutritional status was evaluated by the Mini Nutritional Assessment scale according to exclude the patients with malnutrition. All previous demographic characteristics were evaluated under resting conditions at the beginning of the study. While other measurements, spatiotemporal gait parameters and postural stability indices were done at baseline (pre) and after 12 weeks of interventions (post).

### Evaluation of spatiotemporal gait parameters

Gait parameters were measured using the GAITRite Walkway System (GAITRite, CIR Systems, Sparta, NJ, USA). GAITRite is a 4.5-m computerized carpet with embedded sensors that activated when mechanical pressure is applied. Sensor activation timing and relative sensor distances were detected by the walkway. After that, the GAITRite version 4.7 application software analyzes spatial and temporal gait parameters for each footfall, as well as an overall range for each parameter. The system has been evaluated and proved to have high test-retest reliability. ^13^ It was used in a previous DPN study. ^14^ Each evaluation takes about 15 min to complete, which includes gait testing and data analysis using the manufacturer's software. The patients were tested at their comfortable walking speed. Manually eliminated footfalls that were only partially captured. The average statistics from three walking trials were used in the analysis. Subjects were told to begin walking two meters before the edge of the mat and continue walking two meters beyond the end of the mat to avoid acceleration and deceleration changes. ^15^ In this study, the therapist assessed step length (cm), step time (sec), double support time (sec), velocity (cm/sec), and cadence (steps/min).

### Evaluation of postural stability indices

Postural stability indices were measured using the Biodex Balance System (BBS) (Biodex Medical System, Shirley, NY, USA). For evaluating balance, the BBS is a valid and reliable tool. The device includes a movable circular platform with a 20° tilt in a 360° range, as well as a computer software interface for objective balance testing and simultaneous movement in the anterior-posterior and medio-lateral directions. BBS provides a numerical stability index (SI) that displays postural instability around the body's center of gravity. The SI illustrates the patient's ability to control the platform's tilting angle and amount of motion. A lower SI score suggests more balance and stability, whereas a high SI score indicates higher movement, less stability, and a considerable deviation. The system provides 12 levels of stability, with level 1 allowing the most tilting and level 12 allowing the least tilting. One of the balance indices that's been measured was the antero-posterior stability index (APSI), medio-lateral stability index (MLSI), and overall stability index (OSI).

All patients were given an explanation of the evaluation procedure, as provided in the BBS operation manuals, prior to the postural stability test. Each patient was instructed to stand barefoot in the center of the locked platform and shift his feet to a position that would allow him to retain the cursor on the visual feedback screen in the center of the screen grid. The patient was told to hold his feet in that posture until the platform could be stabilized once he was in a centered position. The angles of the feet and heel coordinates were then recorded on the platform. These angles were entered into the BBS, and the test started. In order to keep the pointer centered when the platform got unstable, the patient was told to retain his focus on the screen. After that, the computer analyzes the patient's sway motions and reports on his or her ability to control platform variation while in a balanced position. A printout report, including APSI, MLSI, and OSI data was obtained at the end of each test trial. Three trials with one minute of rest between them were obtained for each patient, with the average of these trials being utilized for statistical analysis. ^16^

### Treatment interventions

**Traditional physical therapy program**

Both groups received the same traditional physical therapy program for 12 weeks, three times a week, for 30 min each. The treatment session includes a set of exercises aimed at improving muscle strength, balance, and physical endurance, as well as specific gait training. Each session comprises three phases: warm-up, actual training, and cool-down. Five mins of gentle stretching exercises for the calf, hamstring, quadriceps, and iliopsoas muscles were included in the warm-up activities. The active phase lasted 20 min and was performed on a balance training mat with a high elasticity. Gait training including walking in all directions, weight shifting exercises, balance training exercises on the mat and balance board, and proprioceptive exercises in an open and closed chain were all part of the training phase. After the exercise, there was a 5-minute cool-down phase. During the cool-down phase, patients did deep breathing and static back extensor exercises in a reclined position. ^16^ All of the patients were allowed to proceed with their usual leisure activities. They were asked to report any symptom or feeling of falling during the exercise session.

**Antigravity treadmill training**

An AlterG treadmill (AlterG Pro 200, Alter G Inc, USA) was used to provide antigravity treadmill training to the patients in the experimental group. The AlterG allows the patient to change their body weight from 20% to 100% in 1% increments. The air pressure inside the LBPP chamber can be adjusted from 0 to 2.0 kPa above atmospheric pressure. By allowing full range of motion of the upper and lower body as well as a normal gait mechanism, the LBPP treadmills improve balance and strength, maintain patients in position, support patients laterally, and prevent falls. They are very comfortable to train in for long periods of time and have simple controls for adjusting body weight, speed, and inclination. The AlterG treadmill is comprised of two compressors, a protective transparent chamber, safety locks, and several sizes of pants. The treadmill is linked to the compressors, which employ a controller to control the pressure inside the chamber. The transparent material of the chamber allows the therapist to observe the patient walk on the treadmill and provide him with necessary feedback. The AlterG system's force plate was used to determine the patient's weight. The body weight support was set at 75% of body weight for each patient in this study, with a start speed of 0.1 m/sec. The treadmill speed was gradually increased until each patient's maximal walking capacity was reached without losing control of their lower limbs or treadmill track. For 12 weeks, a 30-minute gait training program was performed three times per week. As a warm-up, patients walked back and forth across the room for around five minutes. The inclination on the treadmill was set to zero degrees. The patients were told to maintain an upright posture on the treadmill belt with their feet flat on the belt, and they could stop the training by pressing the stop button. ^12^

### Statistical analysis

Data were tested for normality using the Shapiro–Wilk test. Data were presented as mean ± SD. Mean changes in gait parameters and postural stability indices dependent variables were analyzed using paired t-test to test within-groups comparisons. Between-groups comparisons were analyzed using unpaired t-test. The level of significance was set at P<0.05. Statistical analysis was performed using SPSS software (version 16.0; SPSS Inc, Chicago, IL) (RRID:SCR 019096).

## Results

A total of 82 elderly Saudi men diagnosed with DPN were selected as potential participants for this study ( Figure 1). 37 of the participants were excluded (29 not meeting the inclusion criteria, and eight refused to participate). This study included 45 men who were randomly assigned into two groups. There were no significant differences in mean age, height, BMI, diabetes duration, FBG, HbA1c, HR, systolic and diastolic blood pressure and nutritional status between experimental and control group (P>0.05) as shown in Table 1. Exercise compliance was 100% for all subjects after therapy.

**Table 1.** The demographic characteristics of participants in both groups (Mean ± SD).

Variables	Experimental group (n = 23)	Control group (n = 22)	T value	P value ^☼^
**Age (year)**	68.09 ± 3.58	67.68 ± 2.5	0.19	0.66 **
**Height (m)**	1.6 ± 0.03	1.61 ± 0.02	0.84	0.37 **
**Weight (kg)**	75.87 ± 7.28	78.27 ± 6.73	1.32	0.26 **
**BMI (kg/m** ^**2**^ **)**	29.59 ± 2.75	30.28 ± 2.92	0.67	0.42 **
**Diabetes Duration (year)**	14.22 ± 1.93	14.96 ± 2.03	1.56	0.22 **
**FBG (ml/L)**	168.43 ± 8.12	166.68 ± 6.99	0.6	0.44 **
**HbA1c (%)**	8.22 ± 0.47	8.17 ± 0.43	0.11	0.74 **
**Resting heart rate (beat/min)**	74.74 ± 3.96	73.86 ± 4.96	0.43	0.52 **
**Systolic blood pressure (mmHg)**	140.78 ± 4.01	140.41 ± 3.67	0.11	0.75 **
**Diastolic blood pressure (mmHg)**	85.26 ± 1.96	85.27 ± 1.78	0.01	0.98 **

BMI: body mass index, HbA1c: glycosylated hemoglobin %, FBG: fasting blood glucose.

^☼^ Level of significance at P<0.05.

* Significant.

** Non-significant.

The mean values of gait parameters (step length (cm), step time (sec), double support time (sec), velocity (cm/sec), and cadence (steps/min) between the experimental and control groups were not significantly different at baseline (P>0.05) ( Table 2). The mean values of gait parameters obtained at the baseline and post-treatment assessments were significantly different in the experimental and control group (P<0.05). The patients in the experimental group exhibited improvements in their gait parameters compared with patients in the control group ( Table 2).

**Table 2.** Pre and post-treatment mean values of gait parameters within each group and between groups.

Variables		Experimental group	Control group	T value	P value ^☼^
**Step length (cm)**	**Pre**	52.04 ± 1.58	51.59 ** ± **1.94	0.74	0.395 **
	**Post**	61.30 ** ± **2.53	56.14 ** ± **1.61	66.07	<0.0001 *
	**T value**	-27.67	-12.27		
	**P value**	<0.0001 *	<0.0001 *		
**Step time (sec)**	**Pre**	0.63 ** ± **0.03	0.62 ** ± **0.03	0.65	0.423 **
	**Post**	0.49 ** ± **0.02	0.55 ** ± **0.04	38.79	<0.0001 *
	**T value**	29.48	15.45		
	**P value**	<0.0001 *	<0.0001 *		
**Double support time (sec)**	**Pre**	0.33 ** ± **0.02	0.32 ** ± **0.03	1.07	0.305 **
	**Post**	0.25 ** ± **0.28	0.29 ** ± **0.03	20.89	<0.0001 *
	**T value**	19.91	14.02		
	**P value**	<0.0001 *	<0.0001 *		
**Velocity (cm/sec)**	**Pre**	71.61 ** ± **2.93	70.90 ** ± **2.76	0.68	0.415 **
	**Post**	83.09 ** ± **3.68	75.73 ** ± **3.49	47.26	<0.0001 *
	**T value**	-33.41	-12.09		
	**P value**	<0.0001 *	<0.0001 *		
**Cadence (steps/min)**	**Pre**	83.87 ** ± **6.76	82.73 ** ± **5.79	0.37	0.547 **
	**Post**	99.78 ** ± **7.37	88.14 ** ± **6.28	32.43	<0.0001 *
	**T value**	-24.48	-8.83		
	**P value**	<0.0001 *	<0.0001 *		

^☼^ Level of significance at P<0.05.

* Significant.

** Non-significant.

The mean values of the overall stability index, anteroposterior stability index, and mediolateral stability index between the experimental and control groups were not significantly different (P>0.05) at the beginning of the study ( Table 3). Between the baseline and post-treatment examinations, there was a significant difference (P<0.05) in the mean stability indices. When compared to patients in the control group, patients in the experimental group showed improvements in postural stability indices ( Table 3).

**Table 3.** Pre and post-treatment mean values of postural stability indices within each group and between groups.

Variables		Experimental group	Control group	T value	P value ^☼^
**Antero-posterior Stability Index (API)**	**Pre**	0.57 ± 0.17	0.62 ** ± **0.14	1.36	0.251 **
	**Post**	0.23 ** ± **0.01	0.55 ** ± **0.10	211.9	<0.0001 *
	**T value**	9.49	5.01		
	**P value**	<0.0001 *	<0.0001 *		
**Medio-lateral Stability Index (MLI)**	**Pre**	0.28 ± 0.09	0.34 ** ± **0.12	3.17	0.082 **
	**Post**	0.14 ** ± **0.04	0.31 ** ± **0.10	49.35	<0.0001 *
	**T value**	9.46	4.41		
	**P value**	<0.0001 *	=0.0002 *		
**Overall Stability Index (OSI)**	**Pre**	0.65 ** ± **0.23	0.75 ** ± **0.16	2.79	0.102 **
	**Post**	0.32 ** ± **0.08	0.70 ** ± **0.15	102.6	<0.0001 *
	**T value**	9.44	10.09		
	**P value**	<0.0001 *	<0.0001 *		

^☼^ Level of significance at P<0.05.

* Significant.

** Non-significant.

## Discussion

The findings of this study showed that a program combining antigravity treadmill training and traditional physical therapy improved gait parameters and balance performance more effectively than a traditional physical therapy program alone. After 12 weeks of treatment, both groups showed improvements in all measured variables. In the experimental group, however, there was a greater improvement.

The use of an antigravity treadmill, which is a device that adjusts the gravity experienced in the lower extremities during walking training by using a unique air pressure control system, resulted in significant improvements in gait parameters and postural stability in the experimental group. This reduces the patient's weight by 80%, allowing them to walk and run without having to bear their full weight. This device is also safe and effective for postoperative rehabilitation because of its precision control of up to 1% of body weight, allowing patients with lower limb injuries to rehabilitate without pain. ^17^ Walking distance can be increased while keeping normal walking patterns, and walking activities can be done without affecting ankle and knee joint range of motion. ^18^

The amount of impact on the knee is reduced when walking on an antigravity treadmill. According to a previous study, reducing gravity to 50% of body weight reduced force transmitted to the knee during early rehabilitation and reduced force transmitted to the knee. ^19^ Furthermore, previous study has showed that utilizing an antigravity treadmill for muscular strength and aerobic training enhanced walking and dynamic balance while keeping the same kinetic movement as normal walking and reduced musculoskeletal system strain. ^20^^,^^21^

Antigravity treadmill training is used to prevent quadriceps muscle atrophy and strengthen muscles in patients with femoral fractures. It also allows for partial weight bearing initially, which may help with long-term gait stability. ^22^ Kim *et al.*, who used an antigravity treadmill to treat adults with femoral fractures, found that both groups improved in muscular strength and endurance activities following the intervention. Furthermore, at 60°/s of hip extensor and gluteus muscular activity, antigravity treadmill training improved muscle strength much more than traditional therapy. As a result, gait training by antigravity treadmill compensates for traditional rehabilitation therapies' limitations and provides a rehabilitation plan for patients with femoral fractures to ensure a stable and effective gait. ^23^

Unloading the body weight during walking has the potential to reduce muscular activity as measured by EMG and change the muscle activation pattern. ^24^^–^^26^ Unweighing has also been shown in the literature to produce a reduction in muscle EMG activities that would be muscle specific. Unloading the body weight during running reduced EMG muscle activities in all muscle groups except hip adductors during the swing phase and hamstrings during the stance phase of the running cycle, according to Hunter *et al.*. ^25^ Unweighting has been shown to reduce cardiorespiratory and metabolic demands in previous research. ^27^ Furthermore, cardiorespiratory and metabolic demands increased with increased walking speed and with lower unloading rate. ^28^

Reduced gravity appears to delay chondrogenesis during the very early stages of cell condensation and cell binding, but has a lesser effect on cartilage growth and development at later stages of chondrogenesis, which is more relevant for rehabilitation, according to scientific data. ^29^^,^^30^ Reduced gravity also decreases the extracellular matrix protein content as well as cell density in neocartilage, whereas increasing the ratio of collagen type II to type I expression. ^29^ According to Wolf’s law, a certain load stimulates biological processes to strengthen, whereas overloading has a potentially negative or catabolic effect on tissues. Extrapolating this to antigravity training, it appears that load reduction is associated with the ability to exercise at higher intensities without the risk of negative or catabolic effects. This demonstrates that antigravity treadmills are successful in reducing ground reaction forces and peak knee joint moments in inverse relation to the applied upward body force. ^30^

The antigravity treadmill has been used to treat many diseases. Berthelsen *et al.*, ^21^ for example, conducted a study in 2014 investigating the use of an antigravity treadmill for ambulation training in muscular dystrophy patients. They found that 10-weeks of antigravity treadmill training improved walking capacity and balance statistically when compared to before training without causing muscle damage. ^21^

Positive outcomes of using AlterG in stroke patients with sequelae have been found in terms of walking speed, walking distance, and a reduction in risk of fall. Independence, mobility, and participation have all been linked to gait speed and endurance. ^31^^,^^32^ In a patient with chronic stroke, training on a pressure-controlled treadmill was associated with enhanced gait parameters, reduced fall risk, increased participation, and reduced the self-perceived negative impact. These findings suggest that a pressure-controlled treadmill might be a reasonable alternative to body-weight-supported locomotor training. ^31^ Popp *et al.* compared conventional therapy to antigravity treadmill treatment on chronic stroke patients, reporting a positive response and improvement in gait and endurance in the antigravity treadmill group. ^33^

The antigravity treadmill provides a safe environment where the patient can retrain their gait and improve their balance by receiving positive biofeedback. There have been some case reports of antigravity treadmills having a positive effect in this population, but more research is required. In order to have a positive impact on the population's health, effective treatment approaches must be implemented in routine clinical practice. ^34^

The findings of this study are similar to that of Sukonthamarn *et al.*, ^35^ who found that after a one-month training program, both the intervention and control groups showed improvements in standing balance, motor power, six-minute walk distance, and the functional ambulatory category scale in subacute to chronic hemiparetic stroke patients within one year of onset. Furthermore, ambulation training using an antigravity treadmill in combination with traditional physiotherapy seems to be superior to traditional physiotherapy alone in terms of balance training. Lastly, the antigravity treadmill is a safe rehabilitative medical device that can help patients in achieving better clinical outcomes. ^35^

The significant improvement in gait parameters and postural stability in the experimental group in this study is consistent with the findings of Miura *et al.*, ^36^ who found that LBPP training could effectively increase exercise tolerance and physical performance in elderly patients. ^36^ These findings reflect the safety and ease of use of such rehabilitation equipment that eliminates the risk of falling during aerobic treadmill training sessions. ^37^

AlterG's advantages over the suspension weight-loss system are primarily reflected in the patient's comfort during the weight-loss process. ^38^ The force is evenly distributed throughout the human body's lower portion, and the patient has no sense of pressure. Furthermore, LBPP can improve venous return during walking, which lowers heart rate significantly. ^37^ All of the aforementioned advantages of aerobic exercise are beneficial to patients with osteoarthritis, particularly those with cardiovascular disease and hypertension, promoting patient rehabilitation. ^39^

### Limitations and recommendations

In terms of limitations, the results of this study should be interpreted with caution because it only included male patients in a particular age range, and dietary regimens and routine activities were not properly monitored. Lack of follow-up data, limiting clinical application of our findings to the antigravity treadmill's short-term effects. It's also difficult to isolate the antigravity treadmill's effect in both groups because of the effect of the physical therapy program. The outcomes of this study are promising, but additional trials with fewer inclusion and exclusion criteria would produce more generalizable results. Long-term studies and a comparison to other harness systems are required. Future research on the antigravity treadmill's impact on muscle strength, energy expenditure, and quality of life in DPN patients could be extremely effective for patients.

## Conclusions

Antigravity treadmill training in combination with traditional physical therapy appears to be superior to traditional physical therapy alone in terms of gait and balance training. As a result, the antigravity treadmill has been found to be an effective device for the rehabilitation of DPN patients.

## Data availability

### Underlying data

Figshare: Underlying data for ‘Effects of antigravity treadmill training on gait and balance in patients with diabetic polyneuropathy: A randomized controlled trial’.

The project contains the following underlying data:

• Demographic characteristics raw data: https://figshare.com/s/2990d265a8bea78e4c27 DOI: 10.6084/m9.figshare.17053808
• Gait parameters and postural stability: https://figshare.com/s/197dc8c07d05380056ea DOI: 10.6084/m9.figshare.17054036
• Flow chart of the study: https://figshare.com/s/15128a129f514ba02d92 DOI: 10.6084/m9.figshare.17054045
• Tables: https://figshare.com/s/10ee7a9d111b2c4487ee DOI: 10.6084/m9.figshare.17054057

### Reporting guidelines

CONSORT Checklist: https://figshare.com/s/23df41d9235a4e2f1e5e DOI: 10.6084/m9.figshare.17054060

## Author contributions

**Ashraf Abdelaal**: Conceptualization, Data Curation, Formal Analysis, Funding Acquisition, Investigation, Methodology, Project Administration, Resources, Software, Supervision, Validation, Visualization, Writing-Original Draft Preparation, Writing-Review & Editing.

**Shamekh El-Shamy**: Conceptualization, Data Curation, Formal Analysis, Funding Acquisition, Investigation, Methodology, Project Administration, Resources, Software, Supervision, Validation, Visualization, Writing-Original Draft Preparation, Writing-Review & Editing.
