# Supplementary material for: Effect of Antigravity Treadmill Training on Gait and Balance in Patients with Diabetic Polyneuropathy: A Randomized Controlled Trial
Source: F1000Res. 2022 Oct 26;11:52. Originally published 2022 Jan 17. [Version 3] doi: 10.12688/f1000research.75806.3 (PMC9763767; doi:10.12688/f1000research.75806.3)
Supplement: Peer review history

# Peer review: Reviewer response for version 3

**DOI:** 10.5256/f1000research.139356.r154248

**Published:** 2022-12-19

**Authors:** Sobhy M Aly

**Affiliations:** 1 Department of Biomechanics, Faculty of Physical Therapy, Cairo University, Cairo, Egypt

Thank you for the revised version.

 My comments were fulfilled.

Is the work clearly and accurately presented and does it cite the current literature?

Yes

If applicable, is the statistical analysis and its interpretation appropriate?

Partly

Are all the source data underlying the results available to ensure full reproducibility?

Yes

Is the study design appropriate and is the work technically sound?

Yes

Are the conclusions drawn adequately supported by the results?

Yes

Are sufficient details of methods and analysis provided to allow replication by others?

Yes

Reviewer Expertise:

NA

I confirm that I have read this submission and believe that I have an appropriate level of expertise to confirm that it is of an acceptable scientific standard.

# Peer review: Reviewer response for version 3

**DOI:** 10.5256/f1000research.139356.r154246

**Published:** 2022-11-16

**Authors:** Mariam A. Ameer

**Affiliations:** 1 Physical Therapy and Health Rehabilitation Department, Faculty of Applied Medical Sciences, Jouf University, Sakaka, Saudi Arabia

The comments are not included, even the T values inside the tables are still present.

Is the work clearly and accurately presented and does it cite the current literature?

Partly

If applicable, is the statistical analysis and its interpretation appropriate?

Partly

Are all the source data underlying the results available to ensure full reproducibility?

Partly

Is the study design appropriate and is the work technically sound?

Partly

Are the conclusions drawn adequately supported by the results?

Yes

Are sufficient details of methods and analysis provided to allow replication by others?

Yes

Reviewer Expertise:

Biomechanics and gait analysis

I confirm that I have read this submission and believe that I have an appropriate level of expertise to confirm that it is of an acceptable scientific standard, however I have significant reservations, as outlined above.

# Peer review: Reviewer response for version 2

**DOI:** 10.5256/f1000research.122755.r150521

**Published:** 2022-09-22

**Authors:** Eman T. Mehanna

**Affiliations:** 1 Department of Biochemistry, Faculty of Pharmacy, Suez Canal University, Ismailia, Egypt

In the current work, a clinical trail was conducted to investigate the effect of combining antigravity treadmill training with physical therapy in improving the stability and movement abilities of elderly patients with diabetic polynueropathy. I have some comments that should be considered by the authors: 

 1. The language of the article is overall good. However, many sentences should be corrected or improved.

 Examples: "Despite the fact that current physical therapy therapies can help patients with DPN......". It is inconvenient to use "physical therapy therapies".

 Also in the methods:  "45 elderly Saudi men diagnosed with DPN are recruited from.......". The past tense "were" should be used here instead of "are".

 These are only examples but the whole article should be revised thoroughly in terms of the language.

 2. In the methods, Evaluation of postural stability indices: "the computer analyzes the patient's sway motions and reports on his or her ability to control platform variation"... why using "her" in this sentence? You have only male participants in this study.

 3. In table 1, why did you use F value? This is supposed to be an unpaired t test. The same applies for the comparison between the two groups in tables 2 and 3.

 4. Please indicate the statistical test used for comparison in the figure legend of each figure.

 5. In your comment on the results in tables 2 and 3, you should explain the details of differences. You can't use a brief meaningless sentence like "Between the baseline and post-treatment examinations, there was a significant difference (P<0.05) in the mean stability indices". Explain those significant differences.

 6. "Kim *et al.*, who used an antigravity treadmill to treat adults with femoral fractures, found that both groups improved in muscular strength and endurance activities following the intervention.". What do you mean by "both groups"?

 7. The abbreviation "EMG" was used in the discussion. You should use the full term " Electromyography" before the abbreviation at the first mention.

 8. "There have been some case reports of antigravity treadmills having a positive effect in this population, but more research is required". Which population?

 9. It seems that the discussion was a collection of copied results and conclusions. The discussion should be revised and rephrased to be consistent and relevant.

Is the work clearly and accurately presented and does it cite the current literature?

Partly

If applicable, is the statistical analysis and its interpretation appropriate?

Partly

Are all the source data underlying the results available to ensure full reproducibility?

Yes

Is the study design appropriate and is the work technically sound?

Yes

Are the conclusions drawn adequately supported by the results?

Yes

Are sufficient details of methods and analysis provided to allow replication by others?

Yes

Reviewer Expertise:

Biochemistry and molecular biology

I confirm that I have read this submission and believe that I have an appropriate level of expertise to confirm that it is of an acceptable scientific standard, however I have significant reservations, as outlined above.

# Peer review: Reviewer response for version 2

**DOI:** 10.5256/f1000research.122755.r137112

**Published:** 2022-09-20

**Authors:** Sobhy M Aly

**Affiliations:** 1 Department of Biomechanics, Faculty of Physical Therapy, Cairo University, Cairo, Egypt

Thank you for the revised version. I have only one remaining point of critique:

 Tables - F values still reported in tables although un paired t-test was used for comparison between groups. Please correct this.

Is the work clearly and accurately presented and does it cite the current literature?

Yes

If applicable, is the statistical analysis and its interpretation appropriate?

Partly

Are all the source data underlying the results available to ensure full reproducibility?

Yes

Is the study design appropriate and is the work technically sound?

Yes

Are the conclusions drawn adequately supported by the results?

Yes

Are sufficient details of methods and analysis provided to allow replication by others?

Yes

Reviewer Expertise:

physiotherapy- biomechanics-statistics

I confirm that I have read this submission and believe that I have an appropriate level of expertise to confirm that it is of an acceptable scientific standard, however I have significant reservations, as outlined above.

# Response

**Published:** 2022-10-01

**Authors:** Shamekh El-Shamy

**Affiliations:** Umm Al-Qura University, Saudi Arabia

**Thank for your valuable comments and I appreciate your time and effort. Authors reply to the reviewer’s comment.**

**Comments:**

**Thank you for the revised version. I have only one remaining point of critique:**

**Tables - F values still reported in tables although un paired t-test was used for comparison between groups. Please correct this.**

 Authors correct this typo in the tables.

# Peer review: Reviewer response for version 2

**DOI:** 10.5256/f1000research.122755.r149029

**Published:** 2022-09-20

**Authors:** Mariam A. Ameer

**Affiliations:** 1 Physical Therapy and Health Rehabilitation Department, Faculty of Applied Medical Sciences, Jouf University, Sakaka, Saudi Arabia

The reviewer would like to thank the author for this great effort. However, The following items should be included in the revised manuscript (Major revision) to clarify the way of writing:

**Introduction**

 - The authors should clarify the causes behind making people above the age of 30 the most commonly exposed to diabetes.

 - Instead, the authors mention the structure of the treadmill or its mechanism as it will be explained in detail in the section on material and methods.

 - What is the opinion of the previous literature about the use of antigravity treadmills in DPN? Also, the authors should concentrate on the gaps in the previous literature.  

**Methods**

**Participants**

 - Please mention the specialty of the physiotherapist and his/her years of experience.

 - What are the methods of assessment for DPN?

 - Uncontrolled diabetes? Is this a factor that can affect the results of the study or not? Please clarify this point.

 - T2DM; for the first time please mention the full names of abbreviations

 - Is there any degree of neurological impairment needed from the authors to conduct this study to detect the improvement?

 - What about the type of medication taken by the subjects? Do they affect balance or equilibrium?

**Outcome measures **

- How many times were these parameters detected? Only before the inclusion in the current study or along the study? How are the authors sure that the levels of these parameters were fixed throughout the study?
- Who performed this screening, the physiotherapist or the referred doctor? What was the degree of DN for those patients?

**Evaluation of spatiotemporal gait parameters**

- What about the clothes of the subjects before and after the assessment?

**Evaluation of postural stability indices**

- The BBS is a valid and reliable tool; please add a reference for this information.

**Treatment intervention**

 The traditional physical therapy program

 - What are the number of sets and repetitions for these exercises per session?

**Antigravity treadmill training**

- Why did the authors select this percent? Please provide justification for this point.
- How are the subjects walking back? Please clarify this point.

**Statistical analysis**

 -Why did the authors use the paired and unpaired t-test instead of MANOVA? MANOVA will be sensitive enough to detect the real effect of the intervention within and between groups and the significant results may be insignificant in the MANOVA test.

**Results:**

 - Table 1, 2,3; Why F value, not t value?

**Discussion**:

 - Is the antigravity treadmill representing the real full body weight bearing walking or is the improvement only an instance as the patient will return to walk full weight bearing again? The authors should clarify this argument by explaining the cause behind the balance and spatiotemporal gait variables improvement, especially since the patient didn't use even any assistive devices.

 - This is in femoral fracture instead of nonwalking at all, but the authors compare between the full weight bearing walking and partial walking what are the behind causes of improving these parameters from full weight bearing waking and partial walking?

 - So the results of the current study return to the improvement of cardiorespiratory demands or decreasing muscle activity. Please clarify this point.

 - This part is not related to the current study, it is preferable to remove it “Reduced gravity appears to delay chondrogenesis during the very early stages of cell condensation and cell binding, but has a lesser effect on cartilage”.

 - It is not related to DN. Dystrophy is completely different and patients may walk independently or with assistive devices, so partial weight-bearing may improve their walking gradually till reach to full weight bearing but the selected subjects in the current study is already walking with full weight bearing without assistive devices.

 - Chronic hemiparetic; are the selected subjects walking with assistive devices or not? What is the percent of weight used during training? Was it similar to the current study?

 - Improve venous return; is this improvement instance and returned again after complete weight bearing in a normal situation?

Is the work clearly and accurately presented and does it cite the current literature?

Partly

If applicable, is the statistical analysis and its interpretation appropriate?

Partly

Are all the source data underlying the results available to ensure full reproducibility?

Partly

Is the study design appropriate and is the work technically sound?

Partly

Are the conclusions drawn adequately supported by the results?

Yes

Are sufficient details of methods and analysis provided to allow replication by others?

Yes

Reviewer Expertise:

Biomechanics and gait analysis

I confirm that I have read this submission and believe that I have an appropriate level of expertise to confirm that it is of an acceptable scientific standard, however I have significant reservations, as outlined above.

# Peer review: Reviewer response for version 1

**DOI:** 10.5256/f1000research.79728.r125998

**Published:** 2022-03-16

**Authors:** Sobhy M Aly

**Affiliations:** 1 Department of Biomechanics, Faculty of Physical Therapy, Cairo University, Cairo, Egypt

Thank you for inviting me to review this paper.

 This research studies the effect of antigravity treadmill training on gait and balance in patients with diabetic polyneuropathy. Subjects were divided randomly into study and control groups. The study group received antigravity treadmill training and physiotherapy while the control group received the physiotherapy alone. The results explained improvement of both groups post-treatment with significant difference between groups post-treatment.

 Minor comments:

***Title***

 "Effects" should be changed to "Effect".

***Results***

 In the abstract: no need to mention all these numbers, just mention if there was a significant difference between groups or not.

***Key words***

 "Traditional rehabilitation" is inappropriate.

***Sample size calculation***

 This is inappropriate. Please revise the parameters for calculation, power and effect size.

***Outcome measures***

 Should include only the outcome measures and not the baseline measures.

***Evaluation of spatiotemporal gait parameters***

 "computerized carpet with embedded sensors that activate..." should be changed to "activated".

 Furthermore, why were patients tested at their maximum walking speed? The patients should be tested at their comfortable walking speed.

***Treatment interventions***

 "5 min of..." should be changed to "five".

***Statistical analysis***

 "Between-groups assumptions were analyzed using one-way analysis of variance (ANOVA)" should be changed to "Between-groups comparisons...".

 Statistical analysis is inappropriate. Either use paired and unpaired t-test or mixed model MANOVA. No need for between-groups ANOVA.

***Results***

 "...(P>0.05) between experimental and control group..." should be changed to "...between experimental and control group (P>0.05)..."

 "The mean values of gait parameters obtained at the baseline and post-treatment assessments, however, were significantly different (P<0.05). When compared to patients in the control group, patients in the experimental group showed improved gait parameters (Table 2)." Please rephrase this section to better explain the results within groups and between groups.

Is the work clearly and accurately presented and does it cite the current literature?

Yes

If applicable, is the statistical analysis and its interpretation appropriate?

Partly

Are all the source data underlying the results available to ensure full reproducibility?

Yes

Is the study design appropriate and is the work technically sound?

Yes

Are the conclusions drawn adequately supported by the results?

Yes

Are sufficient details of methods and analysis provided to allow replication by others?

Yes

Reviewer Expertise:

physiotherapy- biomechanics-statistics

I confirm that I have read this submission and believe that I have an appropriate level of expertise to confirm that it is of an acceptable scientific standard, however I have significant reservations, as outlined above.

# Response

**Published:** 2022-03-22

**Authors:** Shamekh El-Shamy

**Affiliations:** Umm Al-Qura University, Saudi Arabia

**Thank for your valuable comments and I appreciate your time and effort. Authors reply to the reviewer’s comments point by point.**

**Minor comments:**

**Title**

**"Effects" should be changed to "Effect".**

 Author changed it to “Effect”.

**Results**

**In the abstract: no need to mention all these numbers, just mention if there was a significant difference between groups or not.**

 These numbers showed the significant improvement after the treatment. 

**Key words**

**"Traditional rehabilitation" is inappropriate.**

 Author changed it to “Traditional treatment”.

**Sample size calculation**

**This is inappropriate. Please revise the parameters for calculation, power and effect size.**

 Author has revised the parameters and corrected it.

**Outcome measures**

**Should include only the outcome measures and not the baseline measures.**

 Author includes these measures to follow up the diabetic patients during the study.

**Evaluation of spatiotemporal gait parameters**

**"computerized carpet with embedded sensors that activate..." should be changed to "activated".**

 Author corrects it “activated”

**Furthermore, why were patients tested at their maximum walking speed? The patients should be tested at their comfortable walking speed.**

 Author corrects it to “comfortable”.

**Treatment interventions**

**"5 min of..." should be changed to "five".**

 Author changes it to “five”.

**Statistical analysis**

**"Between-groups assumptions were analyzed using one-way analysis of variance (ANOVA)" should be changed to "Between-groups comparisons...".**

 Author changes it to "Between-groups comparisons...".

**Statistical analysis is inappropriate. Either use paired and unpaired t-test or mixed model MANOVA. No need for between-groups ANOVA.**

 Author used paired and unpaired t-test.

**Results**

**"...(P>0.05) between experimental and control group..." should be changed to "...between experimental and control group (P>0.05)..."**

 Author changes it to “...between experimental and control group (P>0.05)..."

**"The mean values of gait parameters obtained at the baseline and post-treatment assessments, however, were significantly different (P<0.05). When compared to patients in the control group, patients in the experimental group showed improved gait parameters (Table 2)." Please rephrase this section to better explain the results within groups and between groups.**

 Author rephrases this section

 The mean values of gait parameters obtained at the baseline and post-treatment assessments were significantly different in the experimental and control group (P<0.05). The patients in the experimental group exhibited improvements in their gait parameters compared with patients in the control group (Table 2).
